# Identification of *ANLN* as a new likely pathogenic gene of branchio‐otic syndrome in a three‐generation Chinese family

**DOI:** 10.1002/mgg3.525

**Published:** 2018-12-11

**Authors:** Lisha Deng, Yuanzhen Liu, Wenjun Xia, Jiongjiong Hu, Zhaoxin Ma

**Affiliations:** ^1^ Department of Otorhinolaryngology Shanghai East Hospital, Tongji University Shanghai China; ^2^ Department of Otorhinolaryngology Shanghai East Hospital (Ji’an campus) Ji’an China; ^3^ Institutes of Biomedical Science Fudan University Shanghai China

**Keywords:** *ANLN*, autosomal dominant, branchio‐otic syndrome, exome sequencing

## Abstract

**Background:**

Branchio‐oto‐renal (BOR) syndrome is one of the most common autosomal dominant hearing loss syndromes and features clinical and genetic heterogeneity. When there is no renal deformity, this disease can also be called branchio‐otic (BO) syndrome. Though many genes have been reported, there are still many BO syndrome‐related genes to be identified. To identify a hitherto unknown candidate gene causing BO syndrome in a three‐generation Chinese family, clinical, genetic, and functional analyses were employed.

**Methods:**

Whole‐exome sequencing (WES) was conducted in three affected family members and two unaffected family members. PCR‐Sanger sequencing was performed in all of the family members for segregation analysis and verification of the candidate variants. PCR‐Sanger sequencing was also employed in 150 healthy people to examine the variants. In silico analysis was used to predict possible changes in the protein structure that may affect the phenotype.

**Results:**

We identified a heterozygous missense variant in *ANLN*: NM_018685.4: c.G1105A; NP_061155.2: p.G369R that segregated in the pedigree with an autosomal dominant pattern. No variant was found in the 150 controls and normal family members at this site. The variant c.G1105A was located in a highly conserved F‐actin binding site. The amino acid residue at position 369 in the *ANLN* protein was highly conserved across different species.

**Conclusion:**

In this study, we identified, for the first time, a heterozygous missense variant in *ANLN* (NM_018685.4: c.G1105A; NP_061155.2: p.G369R) that is likely to be a candidate causative gene of BO syndrome in a specific Chinese family.

## 1 INTRODUCTION

Branchio‐oto‐renal (BOR) syndrome (BOR1 MIM#113650; BOR2 MIM#610896) is one of the most common autosomal dominant hearing loss syndromes and affects about one in 40,000 people worldwide and 2% of profoundly deaf children (Fraser, Sproule, & Halal, 1980). BOR syndrome is characterized by varying combinations of branchial, otic, and renal anomalies(Melnick, Bixler, Silk, Yune, & Nance, 1975). Hearing loss is the most commonly observed feature of the syndrome and can be conductive, sensorineural, or a mix of the two (Gimsing & Dyrmose, 1986). If there is no renal deformity, this condition can also be called branchio‐otic (BO) syndrome (BOS1 MIM#602588; BOS2 MIM#120502; BOS3 MIM#608389). In 1997, the human homolog of the Drosophila eyes absent gene *(EYA1 *MIM#601653*)* was reported as the first causative gene for BOR syndrome (Abdelhak et al., 1997). *EYA1* variants can be detected in approximately 40% of persons with BOR syndrome and approximately 20% of those patients carried complex genomic rearrangements of *EYA1 *(Chang et al., 2004). In addition, variants in SIX homeobox 1 gene (*SIX1* MIM#601205) and SIX homeobox 5 gene (*SIX5 *MIM#600963) were found to be related to BOR syndrome (Hoskins et al., 2007; Ruf et al., 2004). Furthermore, Engels, Kohlhase, and McGaughran (2000) and Morisada et al. (2014) successively demonstrated that spalt‐like transcription factor 1 gene (*SALL1 *MIM#602,218) is associated with BOR phenotypes.

Anillin (*ANLN* MIM#616027), an actin‐binding protein, was first identified in *Drosophila* and it plays *a* critical role in cytokinesis (Field & Alberts, 1995; Piekny & Maddox, 2010). In addition to actin, *ANLN* has multiple other binding partners, such as myosin II, septins, and the small GTPase Rho (Kinoshita, Field, Coughlin, Straight, & Mitchison, 2002; Oegema, Savoian, Mitchison, & Field, 2000; Piekny & Glotzer, 2008; Straight, Field, & Mitchison, 2005). These various binding partners imply that *ANLN* acts as an important scaffold for the actin–myosin and microtubule cytoskeletons. *ANLN* can assemble several key components related to cell division during cytokinesis and is regarded as the central organizer (Hickson & O'Farrell, 2008). In 2015, a new role for *ANLN *was found in the control of intercellular adhesion in mammalian epithelial junctions via different mechanisms, including suppression of JNK activity and control of the assembly of the perijunctional cytoskeleton (Wang, Chadha, Feygin, & Ivanov, 2015).

Furthermore, *ANLN* is associated with several diseases. In humans, a missense mutation in *ANLN* was identified as a cause of FSGS (focal segmental glomerulosclerosis; FSGS8, MIM 616032), which is characterized by segmental scarring of the glomerulus and is a leading cause of kidney failure (Gbadegesin et al., 2014). In addition, *ANLN *is up‐regulated in diverse human cancers, including breast, colorectal, endometrial, liver, lung, renal, kidney, ovarian, and pancreatic cancer (Hall et al., 2005). Furthermore, there has also been a report on animal disease conditions*,* suggesting that defective *ANLN* results in abnormal cellular organization in the bronchiolar epithelium, which in turn predisposes the animal to acute respiratory distress (ARDS; Holopainen et al., 2017). However, the underlying role of *ANLN* in BO syndrome has not yet been illuminated.

Here, we describe the identification of a heterozygous missense variant (NM_018685.4: c.G1105A; NP_061155.2: p.G369R) in *ANLN *located in exon 6 in a three‐generation Chinese family with BO syndrome using whole‐exome sequencing, PCR‐Sanger sequencing and in silico analysis. Though the variant has been registered in dbSNP as rs376778595 (https://www.ncbi.nlm.nih.gov/projects/SNP/snp_ref.cgi?rs=376778595), this is the first study that has reported *ANLN* as a likely candidate pathogenic gene for BO syndrome.

## 2 METHODS

### 2.1 Family recruitment and clinical evaluations

#### 2.1.1 Ethical compliance

The Ethics Committee of Shanghai East Hospital, which is associated with Tongji University, approved all of the procedures of this study. And the study was carried out only after written informed consent was obtained from all participants or the parents of subjects younger than 18 years.

#### 2.1.2 Research subjects and controls

**Figure 1:**
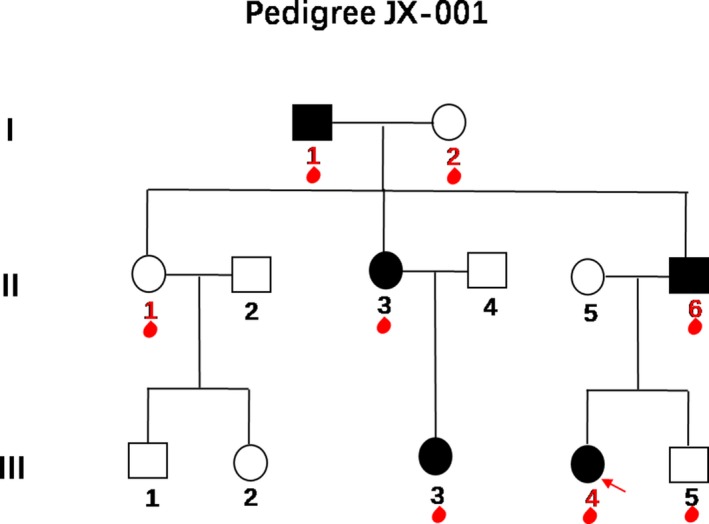
Pedigree of the Chinese family JX‐001 with branchio‐otic (BO) syndrome. In the pedigree, males are denoted by squares; females are denoted by circles. Open symbols denote unaffected individuals; filled black symbols denote affected individuals. The red arrow indicates the proband. Blood drop symbols indicate individuals who donated a blood sample to the study. The numbers in red indicate subjects (I: 1, I: 2, II: 1, II: 6, III‐4) analyzed by WES

This study was conducted in a three‐generation Chinese family presenting as autosomal dominant inheritance (Figure 1). The family consisted of 13 members, including five affected patients diagnosed with BO syndrome according to the diagnostic criteria proposed by Chang et al. (2004). One hundred and fifty normal individuals were selected as controls.

#### 2.1.3 Clinical information

Clinical evaluations were completed by the Department of Otolaryngology and Head and Neck Surgery, Shanghai East Hospital, Shanghai, China. Evaluations included a thorough family history, physical examination, renal ultrasound, bone and air conduction of pure tone audiometry (PTA), computed tomography (CT) scans of the middle ear mastoid, and magnetic resonance imaging (MRI) of the inner auditory and membranous labyrinth.

### 2.2 DNA extraction

A total of 8 members of this family (I‐1, I‐2, II‐1, II‐3, II‐6, III‐3, III‐4, III‐5) and 150 controls were enrolled in this study for the extraction of DNA. Genomic DNA was extracted from 200 ul of whole blood using the QIAGEN‐Blood DNA kit (TIANGENE, Beijing, China) according to the manufacturer's instructions.

### 2.3 Whole‐exome sequencing

Qualified genomic DNA from two unaffected (I: 2 and II: 1) and three affected (I: 1, II: 6 and III‐4) members of this family was utilized for whole‐exome sequencing to systematically search for pathogenic genes. One microgram of purified gDNA was fragmented into 180–280 bp. High‐throughput sequencing was performed on an Illumina HiSeq 2500. Variants that do not meet the following two criteria will be excluded: (a) variants with a frequency of <0.0001 reported in the dbSNP138, HapMap, 1000 Genomes, and other datasets; (b) variants found in all affected individuals (I: 1, II: 6 and III‐4), but not in any unaffected individuals (I: 2 and II: 1).

### 2.4 PCR amplification and Sanger sequencing

To determine whether any of the remaining variants co‐segregated with the disease phenotype in this family, Sanger sequencing was employed to validate the variants in the candidate genes screened with exome sequencing. The sequence‐specific primers (Supporting Information Table S1) flanking the candidate loci were designed online using Primer3 Input (http://primer3.ut.ee/) and synthesized by tsingke, Shanghai, China. All sequences were analyzed with Mutation Surveyor 4.0.8 software and DNAMAN Version 7 software.

### 2.5 In silico analysis

We used SIFT, Polyphen2, and Mutation Taster to predict possible impact of the amino acid residue at position 369 on structure and function of the human protein. Comparisons among the human wild‐type *ANLN *protein sequence and orthologs from *Mus musculus*, *Xenopus laevis*, *Bos taurus*, *Ovis aries*, *Pan troglodytes*, *Pelodiscus sinensis*, *Ficedula albicollis*, *Oryctolagus cuniculus*, and *Felis catus* were conducted online via UniProt (http://www.uniprot.org/) to examine the evolutionary conservation of this protein.

## 3 RESULTS

### 3.1 Clinical description

**Figure 2:**
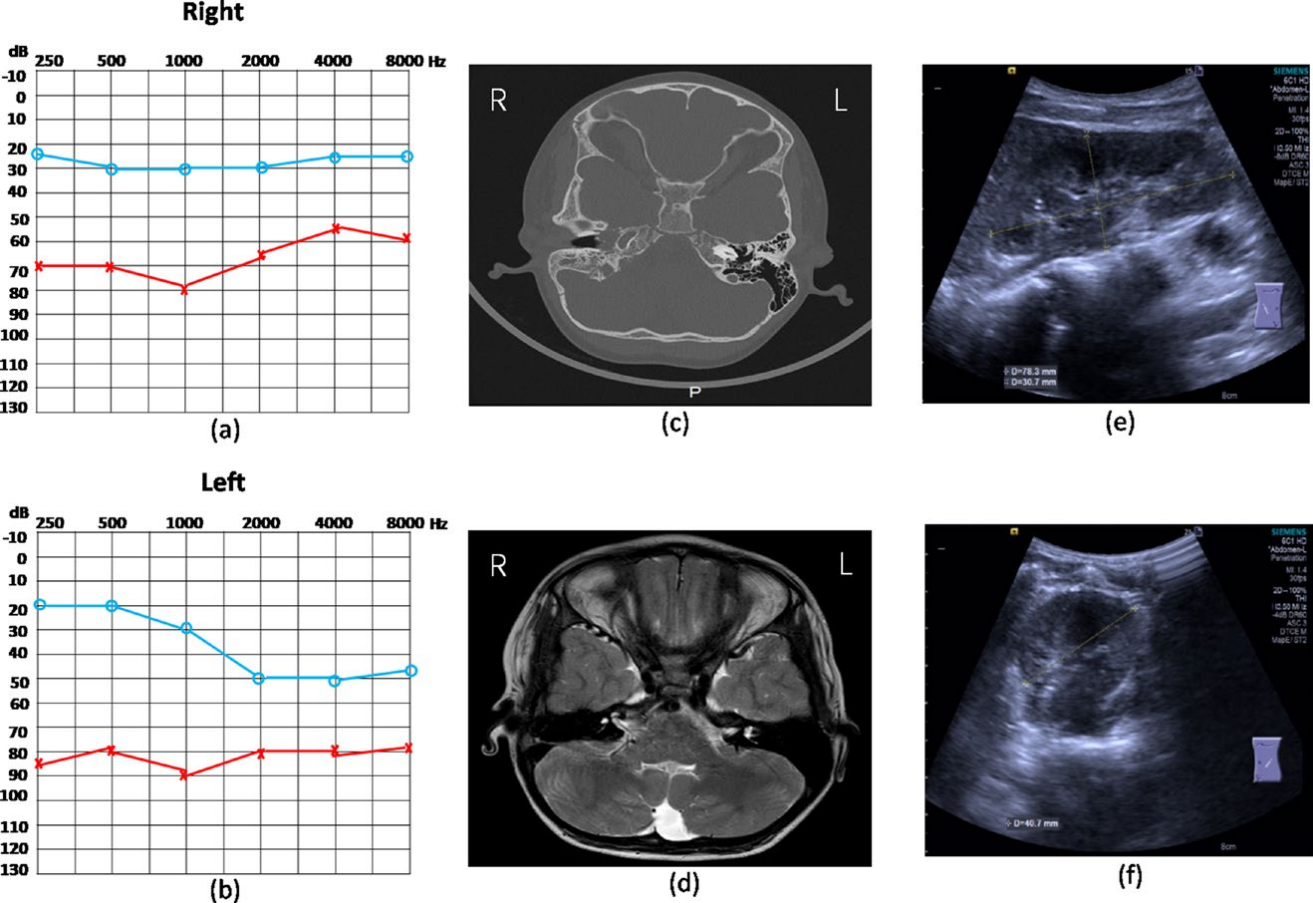
The phenotypes of the proband (III: 4, female, 7 years old) (a, b) Pure tone bone and air conduction thresholds of the right ear (a) and left ear (b) were presented, respectively. Abscissa represents frequency (Hz); Ordinates represent decibel (db). (c) CT scan data showed the ossicular chain malformation in the left ear. (d) Based on the MRI data, the cochlea was well developed. Additionally, both the internal auditory meatus and the membranous labyrinth were well developed. (e, f) Renal ultrasound data showed the right kidney (e) and the left kidney (f) were well developed

The family JX‐001 consists of 13 members, of whom five are BO syndrome patients and the rest have normal phenotypes. The proband manifested with bilateral hearing impairment, bilateral branchial clefts, bilateral preauricular pits, and left middle ear malformation. PTA displayed severe conductive hearing loss of the right ear and severe mixed deafness of the left ear (Figure 2a,b). CT data indicated an ossicular chain malformation in the left ear (Figure 2c). MRI data showed that the cochlea, internal auditory meatus, and membranous labyrinth were all well developed (Figure 2d). Renal ultrasound showed that both the right and the left kidneys were well developed (Figure 2e,f). A summary of the clinical features of the affected family members is shown in Table 1. None of the patients had any history of constant exposure to noise or ototoxic drugs or a history of serious infection during pregnancy.

**Table 1:** Clinical features of the affected family members

Patient	I: 1	II: 3	II: 6	III: 3	III: 4
Gender	Male	Female	Male	Female	Female
Age (year)	59	35	30	6	7
Age of onset (year)	7	21	23	5	5
Second branchial arch anomalies	Bilateral fistule	Bilateral fistule	Left fistule	Bilateral fistule	Bilateral fistule
Deafness	Not specified	Bilateral, moderate, conductive	Bilateral, moderate, mixed	Not specified	Bilateral, severe, mixed
Preauricular pits	Left pits	Bilateral pits	Right pits	Bilateral pits	Bilateral pits
Middle ear anomalies	Unknown	Bilateral ossicular chain deformity	Left ossicular chain deformity	Bilateral ossicular chain deformity	Left ossicular chain deformity

The proband of the JX‐001 family, III: 4, is now 7 years old, had an age of onset of 3 years old, and presents with bilateral anterior ear fistulas, bilateral branchial fistulas, and bilateral moderate‐to‐severe mixed deafness. The onset age of all the other affected members was specified in Table 1. All affected members manifest as single/double ear fistulas, single/bilateral branchial fistulas, and bilateral moderate‐to‐severe mixed or conductive deafness. From the JX‐001 family map, the information obtained can be summarized as follows: the family shows a continuous genetic phenomenon; the incidence ratio of male to female members is 2:3; the proportion of diseased members is equal to the proportion of normal members; the offspring of normal members are normal; at least one of the parents of the patient is affected. Therefore, it can be concluded that the pedigree is an autosomal dominant hereditary family with BO syndrome.

### 3.2 Whole‐exome sequencing

We excluded variants in known genes (*EYA1*, *SIX1*, and *SIX5*) that are causative for BO syndrome in all of the affected individuals by Sanger sequencing. After removal of the variants found in all of the affected individuals (I: 1, II: 6 and III‐4) but not found in any of the unaffected individuals (I: 2 and II: 1), 72 variants segregating with the disease in the five specimens were obtained. Next, variants with frequencies >0.0001 reported in the dbSNP138, HapMap, 1000 Genomes, and other datasets were removed. And the candidate variants were finally reduced to 9 nonsynonymous, heterozygous variants. Specific WES data of the 9 candidate variants were included in Supporting Information Table S2.

### 3.3 A missense variant was found in *ANLN *gene

**Figure 3:**
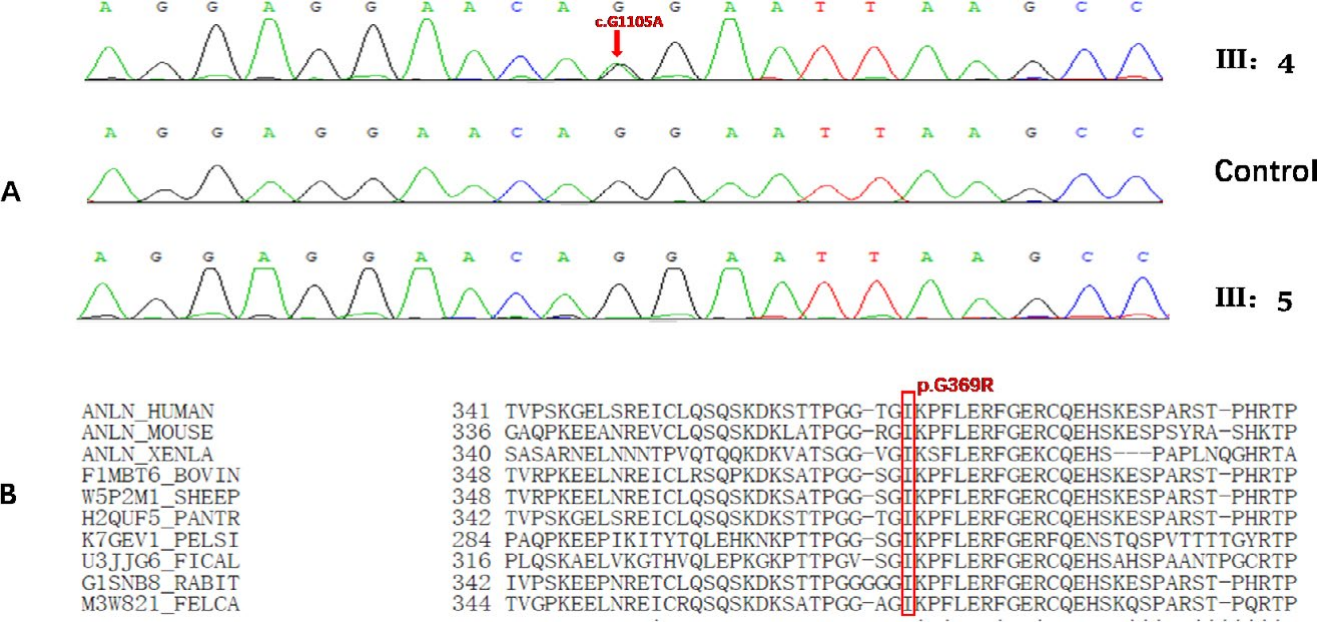
Identification of c.G1105A variant. (a) The variant c.G1105A in *ANLN* gene. Partial sequence chromatograms of *ANLN* gene from an affected individual (III: 4) and a normal individual of the family (III: 5), and a control. The arrow indicates the location of the nucleotide changes at position 1105. (b) Protein alignment shows high conservation of the p.G369R (shown with red squares) in healthy *Homo sapiens*, *Mus musculus*, *Xenopus laevis*, *Bos taurus*, *Ovis aries*, *Pan troglodytes*, *Pelodiscus sinensis*, *Ficedula albicollis*, *Oryctolagus cuniculus*, and *Felis catus*

Subsequently, we screened the above nine variants among all of the pedigree samples by Sanger sequencing and we found a missense variant in exon6 of *ANLN*: NM_018685.4: c.G1105A; NP_061155.2: p.G369R that co‐segregated with the disease (Figure 3a). That is, this *ANLN *variant was exclusively found in all five affected members but was not found in the remaining unaffected family members. In addition, the variant was not found in the 150 controls. And the remaining candidate gene variants were found not co‐segregated with the disease (Supporting Information Figure S1). Thus, our data suggested that the heterozygous variant c.G1105A in *ANLN* was a likely candidate disease‐causing variant in the Chinese family (JX‐001) with BO syndrome.

### 3.4 In silico analysis

To assess the potential effect of p.G369R on *ANLN* function, we conducted in silico analyses. This variant was predicted to be “Deleterious,” “Probably Damaging,” and “Disease‐causing” by SIFT, Polyphen2, and Mutation Taster, respectively. Conservation analysis indicated that the Pro residue at 369 in the *ANLN* protein was conserved across *Homo sapiens*, *Mus musculus*, *Xenopus laevis*, *Danio rerio*, *Bos taurus*, *Ovis aries*, *Pan troglodytes*, *Cavia porcellus*, *Pelodiscus sinensis*, *Ficedula albicollis*, *Oryctolagus cuniculus*, and *Felis catus* (Figure 3b). This finding suggests that this variant may be the cause of BO syndrome in this Chinese pedigree.

## 4 DISCUSSION

Next‐generation sequencing (NGS) technology, which includes single‐gene tests, gene panels, and exome sequencing or genome sequencing, has become an effective tool for detecting point mutations, copy number alterations and changes in gene expression (Kunz, Dannemann, & Kelso, 2013; Lappalainen et al.., 2013; Yap et al., 2014). Exome sequencing has the advantage of being unbiased in selecting genes for analysis (Gilissen, Hoischen, Brunner, & Veltman, 2011, 2012).

Here, through the use of WES, we identified a variant (*ANLN*: NM_001284301.2: c.G1105A; NP_001271230.1: p.G369R) as a new likely disease‐causing mutation in an autosomal dominant hereditary BO syndrome pedigree. Several findings support our conclusion. First, the heterozygous missense variant c.G1105A was the only coding change that completely co‐segregated with the disease. Second, *ANLN *is a relevant functional candidate gene. Because it acts a role in the assembly of intercellular junctions and in cell division (Gbadegesin et al., 2014; Oegema et al., 2000; Piekny & Glotzer, 2008). Finally, p.G369R is located in a highly conserved F‐actin binding site, disturbing the function of the protein. Hence, this report has significance implications since it uncovers a novel candidate gene for BO syndrome.

*ANLN*, a conserved multi‐domain protein, is a prime candidate for functioning in scaffolding and organizing the cytoskeleton due to its many protein–protein interactions. In humans, *ANLN* is required for cortical polarity and cytokinesis (Beaudet, Akhshi, Phillipp, Law, & Piekny, 2017). Considering that human *ANLN* is normally degraded after mitotic exit and sequestered in the nucleus during interphase, its over‐expression may disturb these normal regulatory mechanisms, freeing *ANLN* to affect the actomyosin cytoskeleton during events outside of cytokinesis, including, cell motility which promotes cell differentiation and the spatial programing of the inner, middle and outer structures of the ears (Noden & Van de Water, 1992; Zhang & Maddox, 2010). Thus, *ANLN* can be predicted to be involved in regulating morphogenesis of the inner, middle and outer ear, the branchial tubes and the kidney.

*ANLN* also plays key roles in embryonic morphogenesis and the regulation of intercellular junctions in human epithelial cells. Adherens junctions (AJ) and tight junctions (TJ) are key morphological features of differentiated epithelial cells. A major function of TJs is to form the continuous intercellular barrier, which is required to separate the tissue spaces between epithelial cells (Anderson & Van Itallie, 2009). Disruption of AJs causes loosening of cell–cell contacts, leading to disorganization of the tissue architecture (Meng & Takeichi, 2009). Junction‐associated F‐actin senses and transduces mechanical forces to orchestrate the responses of multiple epithelial cells, a function that is critical for epithelial morphogenesis (Yonemura, 2011). Some cases of hydrocephalus and renal aplasia in *ANLN*‐mutant, ARDS‐affected Dalmatians were caused by abnormal assembly of intercellular junctions in the epithelium during early organogenesis (Holopainen et al., 2017). Therefore, *ANLN*, an F‐actin binding protein, may figure prominently in the regulation of morphogenesis of the inner, middle and outer ear, branchial tubes and kidneys.

In this study, c.G1105A was found to co‐segregate with the disease and no variant was found in 150 controls and the normal family members at this site. And Holopainen et al. (2017) found that no loss‐of‐function variants were found in 136 unaffected dogs through exploration of the canine variant database. In addition, exploration of the public Genome Aggregation Database revealed four heterozygous frameshifts and fourteen heterozygous nonsense variants with very low allele frequencies. The low frequency of pathogenic *ANLN* variants may be because *ANLN *is essential for cell division and because it participates in pathways that are critical during development (Monzo et al., 2005; Zhang & Maddox, 2010). It therefore appears that the loss‐of‐function variants in *ANLN *are extremely rare across species, supporting the critical role of this gene for disease and survival.

Additionally, *ANLN *was associated with other human and animal diseases. Gbadegesin et al. identified a missense mutation in *ANLN* as a cause of focal segmental glomerulosclerosis (FSGS) and found the mutant *ANLN* displayed reduced binding to the scaffold protein CD2AP (Gbadegesin et al., 2014). *ANLN *has also been linked to focal acute respiratory distress syndrome (ARDS). A recent study found that mutant *ANLN* caused abnormal celluar organization of the bronchiolar epithelium, which in turn resulted in ARDS (Holopainen et al., 2017). *ANLN *is over‐expressed in glomeruli affected by FSGS, but not detected in normal glomeruli of humans (Gbadegesin et al., 2014). Interestingly, no renal defect was found in the affected Dalmatians (Holopainen et al., 2017), which is consistent with the result in our study that renal ultrasound of the patients from this family did not reveal any renal malformation. And specific *ANLN* expression was not detected in organs other than lung in the canine. It was supposed that *ANLN* expression is induced in response to podocyte injury and repair, not in the end‐differentiated mature podocyte (Gbadegesin et al., 2014). Thus, it is possible that *ANLN *is not expressed in kidneys of members in this family. Further studies must be carried out to verify the actual expression in the ear, branchial and kidney biopsy samples from normal humans and those affected by BO syndrome.

## 5 CONCLUSION

In this study, we excluded mutations in known BO‐causing genes (*EYA1, SIX1, *and *SIX5*) in this family. Moreover, we found a heterozygous missense variant in exon6 of *ANLN*: NM_018685.4: c.G1105A; NP_061155.2: p.G369R that co‐segregated with BO syndrome in this distinctive family, suggesting a highly conserved role of Pro369 in the human amino acid sequence. Furthermore, our results will help to increase the clinical understanding of BO syndrome induced by variants in this gene. This is the first study that has reported *ANLN* as a likely candidate pathogenic gene for BO syndrome in a three‐generation Chinese family. In the future, experiments on the functional changes of the mutated gene and protein will be performed.

## CONFLICT OF INTEREST

The authors declare that they have no competing interests.
