# Supplementary figures and images for: Identification of ANLN as a new likely pathogenic gene of branchio‐otic syndrome in a three‐generation Chinese family
Source: Mol Genet Genomic Med. 2018 Dec 11;7(2):e00525. doi: 10.1002/mgg3.525 (PMC6393648; doi:10.1002/mgg3.525)

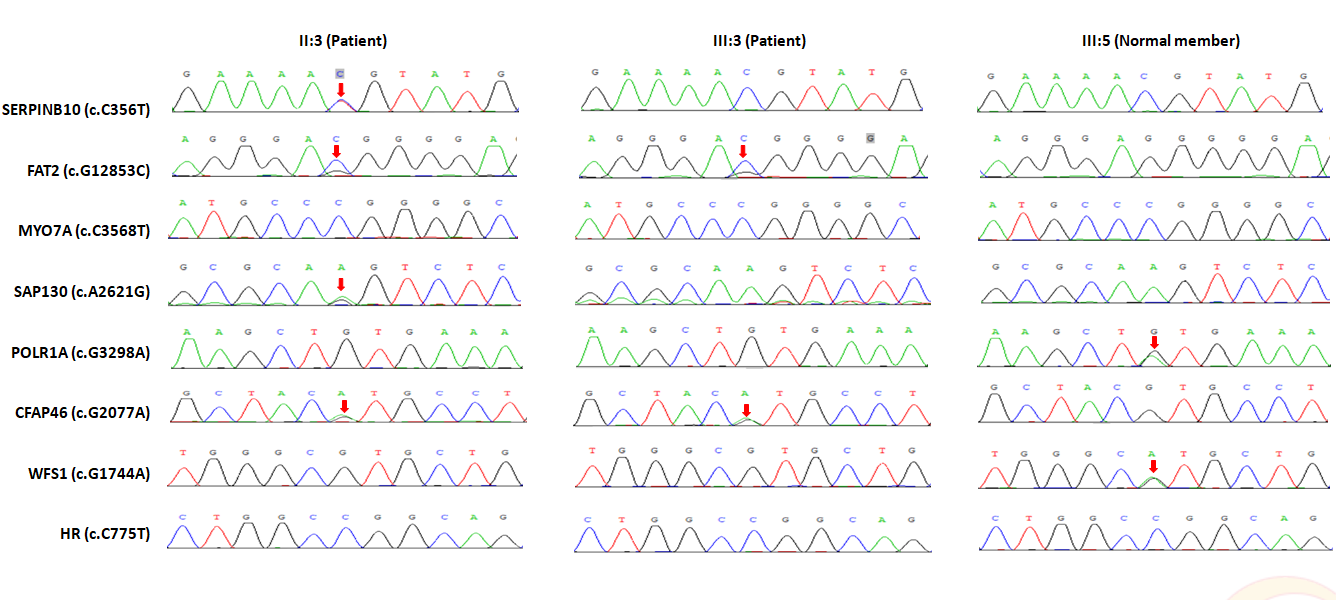

Supplement: Supplementary file 1 [file MGG3-7-na-s001.TIF]
