# Supplementary material for: Identification of ANLN as a new likely pathogenic gene of branchio‐otic syndrome in a three‐generation Chinese family
Source: Mol Genet Genomic Med. 2018 Dec 11;7(2):e00525. doi: 10.1002/mgg3.525 (PMC6393648; doi:10.1002/mgg3.525)
Supplement: Supplementary file 2 [file MGG3-7-na-s002.docx]

**Table S1 Primers for candidate genes**

| **Gene** | **OMIM** | **Genebank reference**  **sequence** | **AA Change** | **Forward primer** | **Reverse primer** |
| --- | --- | --- | --- | --- | --- |
| **SERPINB10** | ***602058** | **NC_0000018.10** | **E3:c.C356T:p.T119M** | **TCCCCACGCCCTTTTAATCT** | **ACAGTCTCCAGCCAAGTCTC** |
| **FAT2** | ***604269** | **NC_000005.10** | **E23:c.G12853C:p.G4285R** | **CTAGCCTTGAGCTTGTCCCA** | **GCACATTTCAAGGGACAACA** |
| **MYO7A** | ***276903** | **NC_000011.10** | **E28:c.C3568T:p.R1190W** | **CCCGATGATCCTGTCTCCAA** | **CTCCCCATACCCCTTCACAG** |
| **SAP130** | ***609697** | **NC_000002.12** | **E16:c.A2621G:p.K874R** | **TAAGGGAGGAGTTTGCAGCT** | **GGGGTTCCGATAGTGACGAA** |
| **POLR1A** | ***616404** | **NC_000002.12** | **E23:c.G3298A:p.V1100M** | **TGTCACGCCCTCATCTCTTT** | **GTCCCCACCTTACATTCCCA** |
| **CFAP46** | **/** | **NC_000010.11** | **E17:c.G2077A:p.V693M** | **TGGCTTTAGTCCCTGGAGTG** | **CAGGACGCTGGAAAAGACAC** |
| **WFS1** | ***606201** | **NC_000004.12** | **E8:c.G1744A:p.V582M** | **CCGTGCCTGCTCTATGTCTA** | **CATGTTGATGGCAGACTCGG** |
| **HR** | ***602302** | **NC_000008.11** | **E3:c.C775T:p.R259W** | **TGTCAGTTCTGCCCATCCAT** | **CCTTGTTCACTTCCTCGCTG** |
| **ANLN** | ***616027** | **NC_000007.14** | **E6:c.G1105A:p.G369R** | **ATGAGTAGGTTTTAGGGCATGT** | **TCTATGCAGAGACCATGTGATG** |

“*” represents a known genetic pattern; “**/”** represents no items were found in http://www.omim.org.
