# Supplementary material for: Identification of ANLN as a new likely pathogenic gene of branchio‐otic syndrome in a three‐generation Chinese family
Source: Mol Genet Genomic Med. 2018 Dec 11;7(2):e00525. doi: 10.1002/mgg3.525 (PMC6393648; doi:10.1002/mgg3.525)
Supplement: Supplementary file 3 [file MGG3-7-na-s003.docx]

|  |  | | | **Table S2** **WES data of the candidate gene** | | | | | | | | | | | | |
| --- | --- | --- | --- | --- | --- | --- | --- | --- | --- | --- | --- | --- | --- | --- | --- | --- |
| **GeneName** | | **CHROM** | **QUAL** | | **FILTER** | **Func** | **ExonicFunc** | **AAChange** | **III:4*** | **II:6*** | **I:1*** | **I:2*** | **II:1*** | **esp6500siv2_all** | **ExAC_ALL** | **ExAC_EAS** |
| **SERPINB10** | | **18** | **196** | | **PASS** | **exonic** | **missense SNV** | **NM_005024:c.C356T** | **0/1:23:6** | **0/1:24:13** | **0/1:35:18** | **/** | **/** | **.** | **7.46E-05** | **0** |
| **FAT2** | | **5** | **222** | | **PASS** | **exonic** | **missense SNV** | **NM_001447:c.G12853C** | **0/1:125:61** | **0/1:166:71** | **0/1:213:112** | **/** | **/** | **.** | **1.66E-05** | **0** |
| **MYO7A** | | **11** | **222** | | **PASS** | **exonic** | **missense SNV** | **NM_000260:c.C3568T** | **0/1:125:68** | **0/1:145:66** | **0/1:132:69** | **/** | **/** | **.** | **8.31E-06** | **0** |
| **SAP130** | | **2** | **222** | | **PASS** | **exonic** | **missense SNV** | **NM_024545:c.A2621G** | **0/1:51:28** | **0/1:51:17** | **0/1:51:22** | **/** | **/** | **.** | **8.24E-06** | **0** |
| **POLR1A** | | **2** | **222** | | **PASS** | **exonic** | **missense SNV** | **NM_015425:c.G3298A** | **0/1:36:20** | **0/1:39:13** | **0/1:35:18** | **/** | **/** | **.** | **8.28E-06** | **0** |
| **CFAP46** | | **10** | **222** | | **PASS** | **exonic** | **missense SNV** | **NM_001200049:c.G2077A** | **0/1:70:28** | **0/1:64:27** | **0/1:87:38** | **/** | **/** | **.** | **7.06E-05** | **0** |
| **WFS1** | | **4** | **222** | | **PASS** | **exonic** | **missense SNV** | **NM_001145853:c.G1744A** | **0/1:166:99** | **0/1:198:94** | **0/1:211:98** | **/** | **/** | **7.70E-05** | **4.95E-05** | **0** |
| **HR** | | **8** | **222** | | **PASS** | **exonic** | **missense SNV** | **NM_005144:c.C775T** | **0/1:129:70** | **0/1:151:63** | **0/1:168:78** | **/** | **/** | **.** | **7.58E-05** | **0** |
| **ANLN** | | **7** | **214** | | **PASS** | **exonic** | **missense SNV** | **NM_018685:c.G1105A** | **0/1:21:10** | **0/1:29:15** | **0/1:29:14** | **/** | **/** | **7.70E-05** | **.** | **.** |

QUAL: The quality value of the variation, the higher the value, the better. It is common in the literature to use a variation of the mass value greater than 20 as the filtration standard.

FILTER: If the site satisfies all filtering conditions, it is marked as PASS (using internationally accepted filtering criteria);

Func: Annotation for the area where the mutation site is located.

ExonicFunc: SNV or InDel variant type of exon region.

AAChang: Amino acid change.

“*” represents GT: DP: DV.  GT is genotype; including 0/0 (Homozygous)，1/1 (Homozygous) and 0/1 (heterozygous). DP is the total number of reads covered; DV is depth of the variant.

esp6500siv2_all: The population allele frequency of the mutation in the ESP6500 database of the National Heart, Lung, and Blood Institute;

ExAC_ALL: The allele frequency of a mutated base at the variant site in all populations.

ExAC_EAS: Allele frequency of mutated bases at this variant site in East Asian populations;

“/” represents no variant at the specific site is detected in the specimen;

 “.” represents no data available.
